# Morphometric Analysis of the Talocrural Joint and Lateral Ankle Ligament Complex in Human Cadavers: A Population-Specific Study

**DOI:** 10.7759/cureus.107822

**Published:** 2026-04-27

**Authors:** Shishir Kumar, Parveen Kumar Sharma, Dhiren K Panda

**Affiliations:** 1 Anatomy, Institute of Medical Sciences & SUM Hospital, Siksha 'O' Anusandhan University, Bhubaneswar, IND; 2 Clinical Anatomy, Dr S. S. Tantia Medical College, Hospital and Research Centre, Sriganganagar, IND

**Keywords:** ankle anatomy, anterior talofibular ligament, cadaver, calcaneofibular ligament, morphometry, talocrural joint

## Abstract

Background: Chronic ankle instability is a common musculoskeletal condition associated with recurrent sprains and functional impairment. Morphological variations in the talocrural joint and lateral ankle ligaments play a significant role in determining joint stability. However, population-specific anatomical data remain limited in the Indian population.

Objective: To evaluate the morphometric characteristics of the talocrural joint and lateral ankle ligaments in cadaveric specimens and establish baseline anatomical reference values.

Methods: This observational cadaveric study was conducted in the Department of Anatomy, Tantia University, Rajasthan, India, using preserved adult human lower limb specimens of Indian origin. Dissection of the ankle region was performed to expose the anterior talofibular ligament (ATFL), calcaneofibular ligament (CFL), and posterior talofibular ligament (PTFL). Morphometric parameters including ligament length, thickness, and attachment characteristics were measured using digital Vernier calipers. Bilateral analysis was carried out. Data were analyzed using descriptive statistics.

Results: The ATFL demonstrated the highest variability in length and thickness, indicating structural predisposition to injury. The CFL showed relatively consistent morphology, while the PTFL was the thickest and most stable ligament. Minor side-to-side variations were observed. The findings establish population-specific morphometric ranges for lateral ankle ligaments.

Conclusion: Significant morphological variability exists in the lateral ankle ligament complex. These findings provide baseline anatomical data for the Indian population, with implications for clinical diagnosis, surgical planning, and rehabilitation strategies.

## Introduction

The ankle joint, anatomically referred to as the talocrural joint, is a synovial hinge joint that plays a crucial role in locomotion, balance, and weight-bearing activities. It is formed by the articulation of the distal tibia and fibula with the talus, providing controlled dorsiflexion and plantarflexion while maintaining stability during dynamic movements [1].

The stability of the talocrural joint is maintained through a combination of osseous congruity and ligamentous support. The lateral ligament complex, comprising the anterior talofibular ligament (ATFL), calcaneofibular ligament (CFL), and posterior talofibular ligament (PTFL), is particularly important in resisting inversion forces and maintaining joint integrity [2].

Among these, the ATFL is the most frequently injured due to its orientation and biomechanical properties [3]. Variations in ligament morphology, including length, thickness, and attachment sites, may influence susceptibility to injury and contribute to chronic ankle instability [4].

Cadaveric studies provide direct and accurate assessment of ligament morphology, allowing precise measurement of anatomical parameters that are difficult to obtain through imaging alone [5]. Despite extensive studies in Western populations, there is a lack of comprehensive morphometric data specific to the Indian population [6].

Therefore, the present study aims to evaluate the morphological characteristics of the talocrural joint and lateral ankle ligaments in cadavers and to establish baseline anatomical reference values relevant to the Indian population.

## Materials and methods

This observational cadaveric study was conducted in the Department of Anatomy, Tantia University, Rajasthan, India, following approval from the Institutional Ethics Committee (Approval No.: TU/IEC/2023/83). The study was performed over a defined study period using cadaveric specimens obtained from the institutional repository. A total of 60 adult human ankle specimens, comprising 30 right and 30 left sides, were included. Where available, demographic details including sex distribution and age at death were recorded; however, in some specimens, this information was unavailable due to anonymized cadaver records. All specimens were confirmed to be of adult origin and free from prior surgical intervention or gross deformity.

**Figure 1:**
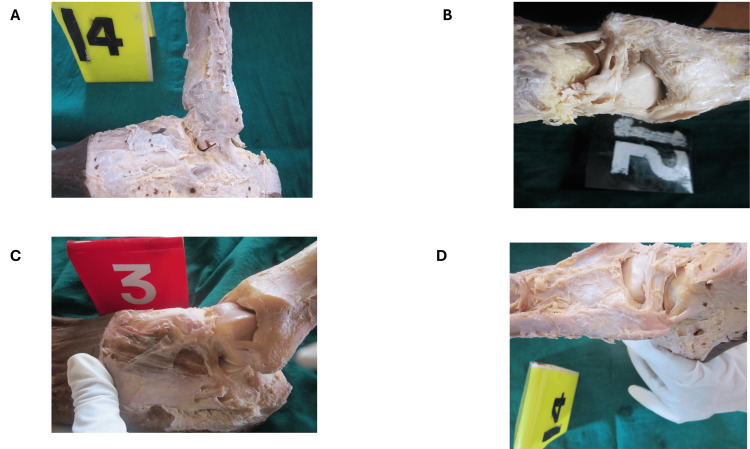
Dissection of lateral ankle ligament complex in cadaveric specimens (A) Lateral dissection view highlighting the ATFL extending from the anterior margin of the lateral malleolus to the talar neck. (B) Posterolateral view of the ankle joint demonstrating the PTFL extending from the posterior aspect of the lateral malleolus to the talus. (C) Lateral view showing the CFL coursing from the tip of the lateral malleolus toward the lateral surface of the calcaneus, with overlying soft tissues partially reflected. (D) Additional lateral dissection illustrating the anatomical relationships of the lateral ligament complex, including the spatial orientation of ATFL and CFL in relation to surrounding structures. ATFL: Anterior talofibular ligament; PTFL: Posterior talofibular ligament; CFL: Calcaneofibular ligament

All specimens were preserved in 10% neutral buffered formalin prior to dissection. A standardized dissection protocol was followed. A longitudinal incision was made over the lateral aspect of the ankle, followed by careful reflection of the skin, superficial fascia, and underlying soft tissues. The peroneal tendons were identified and retracted where necessary to expose the deeper ligamentous structures. The ATFL, CFL, and PTFL were identified based on their anatomical attachments and carefully isolated while preserving their origin and insertion. Representative dissection images demonstrating the anatomical orientation and identification of the lateral ankle ligaments are shown in Figure 1.

**Figure 2:**
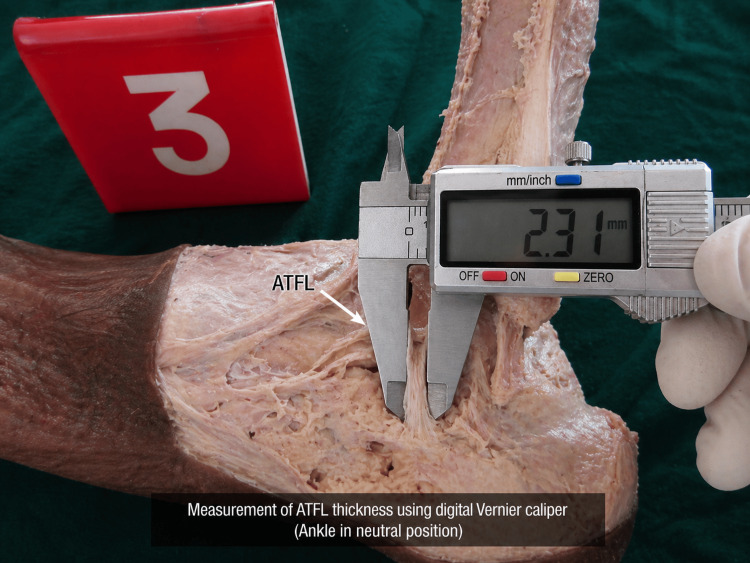
Measurement of ligament thickness using digital Vernier caliper Image demonstrating in situ measurement of ligament thickness using a digital Vernier caliper, with the jaws positioned directly over the ligament to ensure accurate dimensional assessment. The measurement is performed with the ankle maintained in a neutral position, preserving anatomical orientation and minimizing distortion of soft tissues. This technique ensures precision and reproducibility of morphometric data. ATFL: Anterior talofibular ligament

Ligament measurements were performed using a digital Vernier caliper with a precision of 0.01 mm. Measurements were taken in situ to preserve anatomical relationships and avoid distortion. Ligament length was defined as the distance between origin and insertion using defined bony landmarks. For the ATFL, measurements were obtained from the anterior margin of the lateral malleolus to the talar neck; for the CFL, from the tip of the lateral malleolus to the lateral surface of the calcaneus; and for the PTFL, from the posterior aspect of the lateral malleolus to the posterior process of the talus. All measurements were performed with the ankle maintained in a neutral position, approximately 90° between the leg and foot, to standardize ligament tension and reduce positional variability. The measurement technique using a digital Vernier caliper is illustrated in Figure 2.

The ATFL was assessed as a single functional unit for morphometric analysis. In specimens demonstrating a double-banded configuration, measurements were recorded for the overall ligament length rather than individual bands, in order to maintain methodological consistency. All specimens were evaluated for gross anatomical variations, including absence of ligaments or accessory structures; however, no major anomalies were identified, although minor variations such as band differentiation were not separately quantified.

Statistical analysis was performed using SPSS software (version XX). Continuous variables were expressed as mean ± SD, along with 95% confidence intervals where appropriate. The coefficient of variation (CV) was calculated to assess relative variability. Independent samples t-test was used for bivariate comparison between right and left sides. As no statistically significant differences were observed between sides, the data were pooled (n = 60) for inter-ligament comparison. One-way analysis of variance (ANOVA) was used for multivariate comparison among ligaments, followed by post hoc analysis where applicable. A p-value < 0.05 was considered statistically significant.

## Results

A total of 60 ankle specimens (n = 60; 30 right, 30 left) were analyzed. All components of the lateral ligament complex - ATFL, CFL, and PTFL - were successfully identified in all specimens.

Descriptive analysis

The morphometric parameters of the lateral ankle ligaments are summarized in Table 1. The ATFL demonstrated a mean length of 20.5 ± 3.2 mm and thickness of 2.1 ± 0.4 mm, showing the highest variability among the ligaments. The CFL exhibited a mean length of 25.8 ± 2.8 mm and thickness of 2.8 ± 0.3 mm, indicating relatively consistent morphology. The PTFL was the longest and thickest ligament, with a mean length of 28.4 ± 2.5 mm and thickness of 3.5 ± 0.5 mm.

**Table 1:** Pooled morphometric measurements of lateral ankle ligaments (n = 60) CV: Coefficient of variation; ATFL: Anterior talofibular ligament; CFL: Calcaneofibular ligament; PTFL: Posterior talofibular ligament

Ligament	Length (mm, Mean ± SD)	Thickness (mm, Mean ± SD)	Length CV (%)	Thickness CV (%)
ATFL	20.5 ± 3.2	2.1 ± 0.4	15.6	19.0
CFL	25.8 ± 2.8	2.8 ± 0.3	10.9	10.7
PTFL	28.4 ± 2.5	3.5 ± 0.5	8.8	14.3

The CV was calculated to assess relative variability among ligaments. The ATFL demonstrated the highest variability, with CV values of 15.6% for length and 19.0% for thickness, compared to the CFL (10.9% and 10.7%) and PTFL (8.8% and 14.3%), indicating greater structural heterogeneity of the ATFL.

The 95% confidence intervals were narrow across all parameters, indicating reliable estimation of central tendencies. Overall, a graded structural pattern was observed, with PTFL > CFL > ATFL in both length and thickness.

Bivariate analysis

Side-wise comparisons are presented in Table 2. No statistically significant differences were observed between right and left sides for any ligament parameter. The ATFL showed mean length values of 20.8 ± 3.1 mm (right) and 20.2 ± 3.3 mm (left) (p = 0.62). Similarly, CFL measurements were 26.0 ± 2.7 mm versus 25.6 ± 2.9 mm (p = 0.55), and PTFL measurements were 28.6 ± 2.4 mm versus 28.2 ± 2.6 mm (p = 0.48).

**Table 2:** Bilateral comparison of ligament morphometry p-values represent independent sample t-test comparisons between right and left sides. ATFL: Anterior talofibular ligament; CFL: Calcaneofibular ligament; PTFL: Posterior talofibular ligament

Ligament	Side	Length (mm, Mean ± SD)	Thickness (mm, Mean ± SD)	p-value
ATFL	Right vs Left	20.8 ± 3.1 vs 20.2 ± 3.3	2.2 ± 0.3 vs 2.0 ± 0.4	0.62
CFL	Right vs Left	26.0 ± 2.7 vs 25.6 ± 2.9	2.9 ± 0.3 vs 2.7 ± 0.4	0.55
PTFL	Right vs Left	28.6 ± 2.4 vs 28.2 ± 2.6	3.6 ± 0.5 vs 3.4 ± 0.5	0.48

These findings indicate bilateral symmetry of morphometric characteristics. As no statistically significant differences were observed between sides, data from both sides were pooled (n = 60) for subsequent inter-ligament analysis to improve statistical power.

Multivariate (inter-ligament) analysis

Inter-ligament comparisons were performed using one-way ANOVA (Table 3). Statistically significant differences were observed among ligaments for both length (F = 6.82, p < 0.01) and thickness (F = 9.45, p < 0.001).

**Table 3:** Inter-ligament comparison (one-way ANOVA) *Statistically significant (p < 0.05) ANOVA: Analysis of variance

Parameter	F-value	p-value	Interpretation
Length	6.82	< 0.01*	Significant difference across ligaments
Thickness	9.45	< 0.001*	Highly significant difference

Post hoc Tukey analysis demonstrated a graded hierarchy, with PTFL showing significantly greater thickness than both CFL and ATFL (p < 0.01), and CFL demonstrating significantly greater thickness than ATFL (p < 0.05). Similar trends were observed for ligament length.

Post hoc testing indicated a graded pattern, with PTFL greater than CFL and ATFL for thickness, and CFL greater than ATFL for thickness (p < 0.05-0.01).

**Figure 3:**
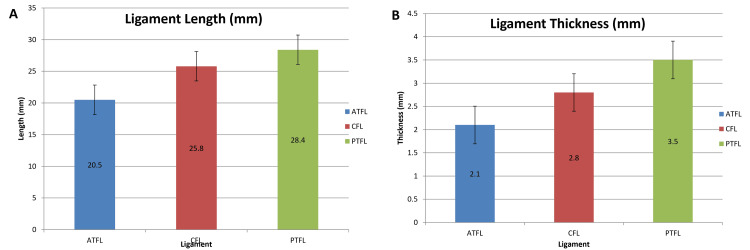
Morphometric comparison of lateral ankle ligaments (A) Bar graph showing mean ligament length (mm) with SD. (B) Bar graph showing mean ligament thickness (mm) with SD. Error bars represent SD.

The morphometric comparison of the lateral ankle ligaments is presented in Figure 1. The morphometric comparison of the lateral ankle ligaments is illustrated in Figure 3, and corresponds exactly to the numerical values presented in Table 1.

Anatomical dissection and ligament identification are demonstrated in Figure 1, while the in situ measurement technique using a digital Vernier caliper is shown in Figure 2, confirming the accuracy and reproducibility of the morphometric methodology.

## Discussion

The present study provides a detailed morphometric evaluation of the lateral ankle ligament complex, demonstrating a clear hierarchical pattern in ligament dimensions, with the PTFL exhibiting the greatest length and thickness, followed by the CFL, and the ATFL showing comparatively smaller dimensions. These findings are consistent with established anatomical descriptions of the lateral ankle ligaments and their structural organization [1,2].

The greater morphometric variability observed in the ATFL in the present study is supported by its known anatomical heterogeneity. Previous studies have demonstrated that the ATFL frequently exhibits a single- or double-banded configuration, with variability in orientation and fiber arrangement [1,3]. In the present study, the ATFL was assessed as a single functional unit, which may contribute to the observed dispersion in measurements. The higher CV for ATFL thickness (19.0%) compared to CFL and PTFL further supports its structural heterogeneity. In contrast, the CFL and PTFL demonstrated lower variability, consistent with their more uniform single-bundle architecture and biomechanical role in stabilizing the ankle joint during inversion and rotational stresses [2,5].

The present findings also highlight differences when compared with previously reported morphometric data from other populations. The mean ATFL length observed in this study (20.5 mm) appears greater than values commonly reported in Western literature, where ATFL length is often cited in the range of approximately 12-15 mm. Such differences may reflect population-specific anatomical variation, as well as methodological factors including measurement techniques, specimen preservation, and definition of ligament boundaries [6,7]. Additionally, variations in ligament morphology may be influenced by genetic, environmental, and functional factors, supporting the importance of region-specific anatomical datasets [7,8]. These findings, therefore, provide context to the “population-specific” characterization of the present study, while acknowledging that direct comparative studies are required for definitive conclusions.

The absence of statistically significant differences between right and left sides is consistent with previous morphometric studies, suggesting bilateral symmetry in ligament dimensions [4,9]. This justified pooling of data for inter-ligament comparison, enhancing statistical power without introducing bias. The statistically significant differences observed among ligaments (p < 0.001) reflect true anatomical variation rather than measurement artifact, further supported by narrow confidence intervals and consistent graphical representation.

From a functional perspective, it is important to note that while the PTFL demonstrated greater thickness and length, these findings should not be directly interpreted as indicators of biomechanical stability. Ligament stability is a dynamic property influenced by multiple factors including fiber orientation, material properties, and joint biomechanics [2,10]. Therefore, the present study provides morphometric insights rather than functional conclusions.

The clinical relevance of these findings is significant. Accurate knowledge of ligament dimensions is essential for anatomical reconstruction procedures such as the Broström technique and its modifications [11-14]. Variations in ligament size and morphology may influence graft selection, surgical technique, and outcomes in patients with chronic ankle instability [15]. Furthermore, understanding morphometric variability, particularly in the ATFL, may aid in improving diagnostic interpretation and surgical planning.

At a neurobiological level, recent studies have demonstrated that ligament injury and instability may be associated with central nervous system adaptations, including altered proprioception and cortical changes [8-10]. These findings further emphasize that ligament morphology and function are interconnected, although the present study focuses on structural parameters.

Several limitations of this study must be acknowledged. First, the use of formalin-fixed cadaveric specimens may result in tissue shrinkage and altered mechanical properties, potentially affecting absolute measurements, particularly ligament thickness. Second, the ATFL was assessed as a single functional unit, and band-specific measurements were not performed, which may limit detailed anatomical characterization. Third, complete demographic data, including sex and age, were not available for all specimens, limiting the ability to perform stratified analysis. Fourth, although no gross anatomical anomalies were observed, subtle variations may not have been fully captured. Finally, while comparative discussion with existing literature has been included, direct population-based comparative analysis was not performed.

Future studies incorporating fresh-frozen specimens, sex- and age-stratified analysis, and detailed classification of ligament morphology are recommended. Additionally, integration of biomechanical testing and imaging-based in vivo assessment may provide a more comprehensive understanding of ligament function.

## Conclusions

Morphometric analysis of the talocrural joint and lateral ankle ligaments reveals significant anatomical variability, particularly in the ATFL. The study provides baseline anatomical data for the Indian population and contributes to improved understanding of ankle joint stability. These findings highlight the importance of individualized anatomical considerations in clinical practice. Incorporating morphometric variability into surgical planning and rehabilitation protocols may improve functional outcomes. Further research integrating imaging, biomechanical testing, and larger population datasets is warranted to enhance clinical applicability.
